# Radiation damage in single-particle cryo-electron microscopy: effects of dose and dose rate

**DOI:** 10.1107/S090904951100820X

**Published:** 2011-04-09

**Authors:** Manikandan Karuppasamy, Fatemeh Karimi Nejadasl, Milos Vulovic, Abraham J. Koster, Raimond B. G. Ravelli

**Affiliations:** a Department of Molecular Cell Biology, Electron Microscopy Section, Leiden University Medical Center, PO Box 9600, 2300 RC Leiden, The Netherlands; b Quantitative Imaging Group, Delft University of Technology, Delft, The Netherlands

**Keywords:** single-particle cryo-electron microscopy, radiation damage, dose, dose-rate effect, macromolecular X-ray crystallography

## Abstract

Radiation damage is an important resolution limiting factor both in macromolecular X-ray crystallography and cryo-electron microscopy. Systematic studies in macromolecular X-ray crystallography greatly benefited from the use of dose, expressed as energy deposited per mass unit, which is derived from parameters including incident flux, beam energy, beam size, sample composition and sample size. In here, the use of dose is reintroduced for electron microscopy, accounting for the electron energy, incident flux and measured sample thickness and composition. Knowledge of the amount of energy deposited allowed us to compare doses with experimental limits in macromolecular X-ray crystallography, to obtain an upper estimate of radical concentrations that build up in the vitreous sample, and to translate heat-transfer simulations carried out for macromolecular X-ray crystallography to cryo-electron microscopy. Stroboscopic exposure series of 50–250 images were collected for different incident flux densities and integration times from *Lumbricus terrestris* extracellular hemoglobin. The images within each series were computationally aligned and analyzed with similarity metrics such as Fourier ring correlation, Fourier ring phase residual and figure of merit. Prior to gas bubble formation, the images become linearly brighter with dose, at a rate of approximately 0.1% per 10 MGy. The gradual decomposition of a vitrified hemoglobin sample could be visualized at a series of doses up to 5500 MGy, by which dose the sample was sublimed. Comparison of equal-dose series collected with different incident flux densities showed a dose-rate effect favoring lower flux densities. Heat simulations predict that sample heating will only become an issue for very large dose rates (50 e^−^Å^−2^ s^−1^ or higher) combined with poor thermal contact between the grid and cryo-holder. Secondary radiolytic effects are likely to play a role in dose-rate effects. Stroboscopic data collection combined with an improved understanding of the effects of dose and dose rate will aid single-particle cryo-electron microscopists to have better control of the outcome of their experiments.

## Abstract 2

The effects of dose and dose-rate were investigated for single-particle cryo-electron microscopy using stroboscopic data collection. A dose-rate effect was observed favoring lower flux densities.

## 1. Introduction

Single-particle cryo-electron microscopy (SP cryo-EM) is a unique technique widely used to elucidate the three-dimensional structures of macromolecules of molecular mass greater than a few hundred kDa (Saibil, 2000 ▶; Frank, 2009 ▶; Jonic & Vénien-Bryan, 2009 ▶; Orlova & Saibil, 2010 ▶). It provides complementary structural information to macromolecular X-ray crystallography (MX) and nuclear magnetic resonance (NMR) techniques which require single crystals and labelled proteins, respectively, as a prerequisite to be studied by such methods. In SP cryo-EM studies numerous projection images are collected from randomly (or sometimes preferentially) oriented macromolecules in a thin layer of a vitreous sample (vitreous being an amorphous state). By computational reconstruction methods, a three-dimensional electron-density map of molecules to a resolution of ∼10 Å (1 nm) can be obtained from these projection images (Frank, 2009 ▶; Wendler & Saibil, 2010 ▶). Further, it is becoming common to achieve pseudo-atomic models of macromolecular complexes to 6–4 Å resolution by fitting the atomic models of some of the components available from X-ray diffraction studies into the reconstructed EM map of the entire complex (for example, Zhou, 2008 ▶; Bhushan *et al.*, 2010 ▶; Sindelar & Downing, 2010 ▶; Baker *et al.*, 2010 ▶; Fujii *et al.*, 2010 ▶). A full-atom model of a non-enveloped aquareovirus at 3.3 Å was recently obtained by SP reconstruction in which side-chain densities for non-Gly amino acids were clearly visible (Zhang *et al.*, 2010 ▶). Technological improvements in electron optics, sample preparation, and data collection and processing have enabled these recent advances.

Radiation damage, unfortunately, will always limit the achievable resolution in SP cryo-EM (Glaeser, 2008 ▶; Massover, 2011 ▶). The damage results from the deposition of energy into the sample owing to the inelastic interactions between the ionizing electron radiation and matter. Traditionally, radiation damage has been treated as a binary nuisance. The total electron flux used to collect SP cryo-EM data is a compromise between the signal-to-noise ratio and the radiation damage. Very high-quality images can be obtained, although at the same time it is usual to discard an unpredictable number of particles for a variety of reasons, such as beam-induced movements (Glaeser, 2008 ▶). At the typical energies used in transmission electron microscopy (TEM), 100–300 keV, inelastic scattering is approximately three times more likely than elastic scattering (Langmore & Smith, 1992 ▶; Henderson, 1995 ▶). Inelastic scattering events include, in order of importance, plasmon scattering, *K*- and *L*-shell ionization, Bremsstrahlung, and fast and slow secondary electron emission. The deposited energy invariably destroys the biological specimen. Studies that describe these effects are as old as cryo-electron microscopy itself (Taylor & Glaeser, 1976 ▶; Glaeser, 2008 ▶).

Radiation damage studies carried out in cryo-EM have received full attention from macromolecular X-ray crystallographers, in particular since radiation damage became a daily nuisance in experiments performed on highly intensive third-generation wiggler and undulator beamlines (reviewed by Ravelli & Garman, 2006 ▶; Garman, 2010 ▶). *Vice versa*, systematic radiation damage studies in MX might be of interest to the SP cryo-EM community. Below, a concise background of relevant studies in MX is given.

The X-ray beam introduces structural changes in the sample during the experiment, resulting in non-isomorphism, which is thought to be a major cause of unsuccessful multiple anomalous dispersion structure determinations (Rice *et al.*, 2000 ▶; Ravelli *et al.*, 2005 ▶). However, by collecting multiple complete data sets within the usable lifetime of a crystal, it has been possible to study radiation damage in unprecedented detail. These studies have been complemented by experimental methods such as UV/VIS microscopy (McGeehan *et al.*, 2009 ▶), fluorescence lifetime microscopy (Royant *et al.*, 2007 ▶), X-ray spectroscopy (Yano *et al.*, 2005 ▶), Raman spectroscopy (McGeehan *et al.*, 2007 ▶; Carpentier *et al.*, 2007 ▶), electron paramagnetic resonance (Utschig *et al.*, 2008 ▶), IR spectroscopy (Sage *et al.*, 2011 ▶) and small-angle X-ray scattering (Meents *et al.*, 2010 ▶), as well as theoretical by simulations (Kuzay *et al.*, 2001 ▶; Kriminski *et al.*, 2003 ▶; Nave & Hill, 2005 ▶; Mhaisekar *et al.*, 2005 ▶).

Radiation damage, in general, can be classified as primary and secondary in nature. The most dominant primary inelastic interaction between X-rays and matter at the energies typically used in MX (8–14 keV) is photoelectric absorption. The atom undergoing photoelectric absorption, typically of the order of 10 per unit cell per synchrotron data set, is a site of primary damage. The energy of the ejected electron depends on the energy of the incoming photon. An emitted photoelectron with ∼12 keV for a 12 keV photon will have a mean free path length of a few micrometers (O’Neill *et al.*, 2002 ▶) and will cause secondary damage due to the excitation and formation of another ∼500 ionization events. The resulting electron-loss and electron-gain centers might cause direct damage to the protein or indirect damage by diffusion through the vitrified cryo-buffer. Diffusible radicals may or may not recombine and might be intercepted by radical scavengers (O’Neill *et al.*, 2002 ▶; Southworth-Davies & Garman, 2007 ▶; Nowak *et al.*, 2009 ▶; Barker *et al.*, 2009 ▶).

Early synchrotron studies of radiation damage in macromolecular crystals at the typical data-collection cryo-temperature (100 K) showed that site-specific damage will occur in a well defined order. Disulfide bonds are in particular susceptible, followed by decarboxylation of aspartate and glutamate residues (Burmeister, 2000 ▶; Ravelli & McSweeney, 2000 ▶; Weik *et al.*, 2000 ▶). The fact that there is a large range in susceptibility among different disulfide bonds and carboxyl groups illustrates the importance of secondary processes. The radical species that are formed upon irradiation of water include hydrogen (H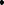) and hydroxyl (OH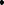) radicals, electrons (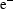) and hydrated electrons (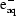). Protons are only known to become mobile in amorphous ice at ∼115 K. OH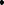 radicals become mobile above 130 K in crystalline ice (Symons, 1999 ▶). Positive holes are rapidly trapped at 77 K (boiling point liquid nitrogen) forming amido radicals on the protein backbone chain, whereas electrons are able to move efficiently at 77 K until they encounter disulfide bonds where they are trapped (Jones *et al.*, 1987 ▶; Ravelli & McSweeney, 2000 ▶; Barker *et al.*, 2009 ▶). The role of secondary processes is temperature dependent; all radicals will gain mobility at higher temperatures but not all radicals can be frozen out at 77 K. Hydrated electrons will still be mobile under helium cooling. At room temperature and neutral pH the yields of hydrated electrons and hydroxyl radicals are approximately equal, while the yield of H atoms is much smaller (Southworth-Davies *et al.*, 2007 ▶). At acidic pH, hydrated electrons rapidly recombine with protons to form H atoms. Both reducing radicals, the hydrated electron and the H atom, react rapidly with oxygen, if present, to yield oxygen-centered radicals that can attack components of the protein. The oxidizing OH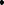 radical is highly reactive and will abstract H atoms from C—H and N—H bonds to form carbon- and/or nitrogen-centered radicals. At room temperature, with many radicals being mobile, an inversed dose-rate effect has been observed and attributed to the increased importance of radical recombination at higher dose rate (Southworth-Davies *et al.*, 2007 ▶). It was shown that OH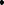 radicals can be effectively scavenged in MX at room temperature (Barker *et al.*, 2009 ▶). Investigations into dose-rate effects in MX at cryogenic temperatures has indicated that such effects are in general small for vitrified samples (Leiros *et al.*, 2001 ▶, 2006 ▶; Ravelli *et al.*, 2002 ▶; Sliz *et al.*, 2003 ▶; Owen *et al.*, 2006 ▶).

The dose in gray (1 Gy = 1 J kg^−1^) can be calculated with the aid of programs such as *RADDOSE* (Murray *et al.*, 2004 ▶, 2005 ▶; Paithankar & Garman, 2010 ▶) from the incident-beam parameters (X-ray flux density, photon energy and beam shape) and the crystal size, together with the absorption and attenuation coefficients obtained from knowledge of the total number of different atom types in the unit cell. The widespread use of dose rather than incident flux density and integration times has greatly facilitated objective comparisons between experiments performed at a large variety of X-ray sources, ranging from sealed tubes to microfocus synchrotron beamlines. The tolerable dose limit for a macromolecular crystal before it loses half of its diffraction intensity, *D*_1/2_, was predicted to be 20 MGy (Henderson, 1990 ▶) based on lifetime measurements on crystalline biological samples in the electron microscope. Owen *et al.* (2006 ▶) experimentally measured a dose limit in MX (*D*_1/2_ = 43 MGy) and recommend a maximum dose of 30 MGy. Others (Kmetko *et al.*, 2006 ▶; Howells *et al.*, 2009 ▶; Holton, 2009 ▶; Holton & Frankel, 2010 ▶) related the fading of the average intensity with dose through a resolution-dependent formula
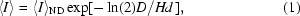
where *D* is the absorbed dose, 〈*I*〉 is the average spot intensity after absorbing a dose *D*, 〈*I*〉_ND_ is the average spot intensity in the absence of radiation damage, ln(2) is the natural log of two, *d* is the resolution in Å, and *H* is a constant (Howells *et al.*, 2009 ▶) of 10 MGy Å^−1^.

Radical recombination has been postulated as a plausible cause for dose-rate effects (Southworth-Davies *et al.*, 2007 ▶). Excessive heating of the sample would also result in a dose-rate effect (Ravelli *et al.*, 2002 ▶). Kuzay *et al.* (2001 ▶) presented a thorough study of the thermal interactions of a cryo-cooled biological sample exposed to an intense strong X-ray beam based on classical heat-transfer theory. The sample is internally heated as the energy of the X-ray beam is absorbed and externally cooled at its surface by convection to a cold N_2_ gas stream. Two theoretical models were presented, a spatially uniform heating of a thin sample for the so-called ‘lumped model’. Here the temperature in the sample is a simple function of time. For thicker samples the temperature will be both a function of time and space; for this a ‘distributed model’ was derived. Kuzay *et al.* showed that heat transfer is limited by the rate of external convection; internal temperature gradients within the crystal are small. Kriminski *et al.* (2003 ▶) refined some of the parameters used in the models described above and concluded that crystal heating by X-ray absorption on present high-flux beamlines should be small (<20 K), although there are new beamlines with flux densities larger than those used in their calculations. Using an IR camera, Snell *et al.* (2005 ▶, 2007 ▶) gave an experimental verification of the calculations of Kriminski *et al.* (2003 ▶) and Kuzay *et al.* (2001 ▶). Glass bead samples were used as a surrogate for the biological samples, and the spatial and temporal distribution of a cryo-cooled glass bead heated by a smaller X-ray beam could be carefully measured and visualized. They confirmed that the heating is not sufficient to raise the sample temperature to the amorphous/crystalline ice transition region of ∼130–140 K (McMillan & Los, 1965 ▶; Weik *et al.*, 2001 ▶).

In this work studies on the effects of dose and dose rate for SP cryo-EM are presented and related to systematic radiation damage studies in MX. The deposited energy per mass unit (dose) used in our SP cryo-EM experiments were estimated from parameters such as flux density, integration time, beam size and energy, protein concentration, sample thickness and the main contribution to inelastic scattering, namely plasmon interaction. The sample thickness was measured using electron tomography. Dose-rate effects were investigated by collecting several series of single-particle data with identical cumulative doses, but with variable incident flux densities and integration times. Analogous to MX, a figure-of-merit (FOM) term is defined to describe the average cosine of phase errors within an aligned image series. It is shown that FOM can be used as a metric for radiation-damage studies. Unlike MX, a clear dose-rate effect could be observed, favoring the use of lower dose rates. Dose-rate effects could originate, as mentioned above, from radical recombination and (or) sample heating. The process of sample heating by the electron beam was studied by simulated systems based on classical heat-transfer models. The potential influence of radical recombination was studied by altering the solvent constituents of the SP sample. High salt and glycerol concentrations, typically used as cryo-protectants in MX, are examined at cryo-temperatures within the TEM to see if they altered the radiation robustness of the sample. Similarly, a low concentration of fixative was used. Results are discussed and compared with recent findings in the literature (Iancu *et al.*, 2006 ▶; Massover, 2007 ▶, 2011 ▶; Chen, Sachse *et al.*, 2008 ▶; Glaeser, 2008 ▶; Bammes *et al.*, 2010 ▶).

## 2. Methods

### 2.1. Experimental methods

#### 2.1.1. Sample preparation

We used *Lumbricus terrestris* erythrocruorin (Hb) as a test sample. This 3.6 MDa extracellular respiratory protein complex, termed either erythrocruorins or hemoglobins (Royer *et al.*, 2000 ▶, 2006 ▶), consists of 144 hemoglobin and 36 linker subunits. The hemoglobin subunits are organized into 12 dodecamers, each of which binds to a heterotrimer of linker proteins. Each dodecamer is a trimer of heterotetramers. The 12 dodecamers form a core complex with *D*_6_ symmetry. The sample was prepared using a protocol adapted from Vinogradov & Sharma (1994 ▶). The harvested concentrated Hb solution was stored at 277 K in 50 m*M* ammonium acetate (measured pH of 6.5) until use. Protein A (a bacterial surface protein commonly used because of its ability to bind immunoglobins) conjugated with 5 nm colloidal gold particles (CMC-UMC, Utrecht, The Netherlands) was added as fiducial markers to the protein sample just before preparation of the EM grids. Aliquots of 3 µl samples at 0.5–1 mg ml^−1^ protein concentration were applied to 200 mesh glow discharged C-flat^TM^ (Protochips Inc., NC, USA) grids (1.2 µm hole size) and blotted from both sides inside an FEI Vitrobot using 3 s blotting time with 100% relative humidity. Subsequently, the blotted grid was rapidly plunged into liquid ethane for vitrification. The grid was stored in liquid nitrogen pending examination in the electron microscope.

In addition to the low-salt control sample described above, three more solvent constituents were tested. The required amount of stock was dissolved to 0.5–1 mg ml^−1^ final protein concentration in (i) 2 *M* ammonium acetate, (ii) 50% (*v*/*v*) glycerol and (iii) 0.2% (*v*/*v*) glutaraldehyde. The sample prepared in 2 *M* NH_4_Ac (as high salt) and 50% (*v*/*v*) glycerol served as a model system for cryo-protectants commonly used in MX. Glutaraldehyde was chosen as it has been used as a stabilizing organic molecule for protein complexes studied in SP cryo-EM (Kastner *et al.*, 2008 ▶; Stark, 2010 ▶). For the glutaraldehyde sample, the protein was incubated in a solution containing 0.2% (*v*/*v*) glutaraldehyde in 50 m*M* ammonium acetate for about 10 min prior to use. Grids were prepared as above.

#### 2.1.2. Image acquisitions/data collection

Images were recorded on a 4k × 4k Eagle on-axis CCD camera using a FEI (http://www.fei.com/) TECNAI Biotwin electron microscope with a LaB_6_ filament operating at 120 kV without using an energy filter. Other microscope settings used were: condenser aperture number 3 (size of 100 µm), objective aperture 3 (70 µm) and spot size index 6. The grid was kept in a Gatan 626 (Gatan Inc., USA) cryo-holder at a temperature of 103 K, as monitored by the temperature control unit. The magnification at the detector plane was ∼68000×, the requested defocus −3 µm and the exposure time 1 s. Images were hardware binned and consist of 2048 × 2048 pixels. The field of view was 0.9 µm × 0.9 µm, the pixel size 4.5 Å square. The incident flux was derived from the detector analog-to-digital units (ADUs) by taking 1 s exposures without sample and using conversion factors (in ADU/e^−^) as calibrated by Vulovic, Rieger *et al.* (2010 ▶) for these systems. Each exposure series was collected from a previously unexposed sample suspended across one of the holes in the C-flat grid. A series of 50 successive images was recorded with an incident flux density of 5 e Å^−2^ s^−1^ (medium flux), corresponding to an integrated flux density for the final images of 250 e Å^−2^. Similarly, a series of 50 images was acquired with an incident flux density of 50 e Å^−2^ s^−1^ (high flux), and another series of 250 images with an incident flux density of 1 e Å^−2^ s^−1^ (low flux). In addition, 50 high-flux images (50 e Å^−2^ s^−1^) were collected with an exposure time of 0.1 s (high-flux short-exposure), resulting in an integrated flux density for the final images of 250 e Å^−2^. The pre-specimen shutter was used for all the experiments: the specimen was only exposed during the data recording. The pre-specimen shutter response of the microscope was checked by comparing the median intensity of the sum of ten images with an exposure time of 0.1 s to the median intensity of one image with 1 s exposure time. The difference was less than 0.09%. All images were collected as fast as possible after each other, resulting in, on average, 13 images per minute.

#### 2.1.3. Sample thickness measurements

In order to calculate the approximate sample thickness, tilt series were acquired and thickness was calculated from the reconstructed tomograms. Single-axis tilt series were recorded using FEI *Inspect3D* software for tilt angles from −52° to +52° in steps of 1° at a detector magnification of ∼68000×, and an incident flux density of 1.3 e Å^−2^ s^−1^. The defocus was set to −5 µm at 0° tilt angle. The *IMOD* software package (Kremer *et al.*, 1996 ▶) was used for data processing and three-dimensional tomographic reconstruction. The approximate sample thickness was derived from the number of sample-containing tomogram slices in the beam direction.

### 2.2. Computational methods

#### 2.2.1. Image alignment

Where relevant, images were corrected for statistical outliers (Vulovic, Rieger *et al.*, 2010 ▶). Account was taken of sample drift by aligning the images to the first image of each series using a normalized cross-correlation function. The translation vectors were calculated with sub-pixel accuracy. The real-space images were translated by applying a corresponding phase shift in Fourier space.

#### 2.2.2. Dose and heat calculations

The dose, in gray (Gy), was calculated based on the incident flux density, exposure time, electron beam size and energy, and the molecular weight and number of Hb particles, in a manner similar to the program *RADDOSE* (Murray *et al.*, 2004 ▶, 2005 ▶; Paithankar & Garman, 2010 ▶). As the product of the dominant form of inelastic electron scattering, only plasmons were taken into account, depositing on average 20 eV per inelastic event into the sample (Langmore & Smith, 1992 ▶).

The temperature rise of the vitreous ice was estimated based on lumped model calculations (Kuzay *et al.*, 2001 ▶). The total deposited energy as determined by the dose calculations was assumed to contribute to heating of the sample. In the ‘lumped system’ the internal temperature spatial variations in the sample are neglected and the temperature changes only with time. The energy balance is given by (Kuzay *et al.*, 2001 ▶)
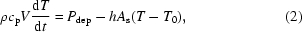
where 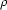 is the density of vitreous ice (0.93 g cm^−3^), *V* is the volume of the illuminated sample, *P*_dep_ is the deposited power (energy per time) to the specimen, *A*_s_ is the area through which heat is conducted, *T*_0_ is the initial temperature of the sample (103 K) and *h* is the heat-transfer coefficient. The heat capacity of the sample (*c*_p_) was taken to be 900 J kg^−1^ K^−1^ (Kriminski *et al.*, 2003 ▶). In a lumped system with isolated walls (adiabatic model) this model predicts a rate of temperature increase of 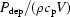 ≃ 61121 K s^−1^ (Fig. 6*a*). This is unrealistic and shows the importance of incorporating the cooling from the ambient and grid into the model. The evolution of the temperature could be written as (Kuzay *et al.*, 2001 ▶)
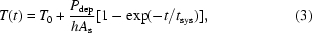
where
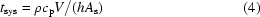
is the system time constant which characterizes the cooling rate. For a short time after the onset of the exposure the system acts like an adiabatic system and the temperature increases linearly with time (Kuzay *et al.*, 2001 ▶). After a time corresponding to three system time constants (3 × *t*_sys_), the sample reaches 95% of the final temperature. If the exposure is shorter than this, the final maximum temperature will not be reached.

In the ‘distributed system’ the temperature is non-uniform both in time and position. The spatial and temporal thermal behavior of the system was simulated as heat diffusion in one dimension from the illuminated spot area to the cryo-cooled copper grid.

The temperature distribution is derived from the diffusion equation,
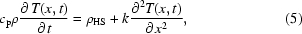
where *k* is the thermal conductivity of vitrified ice. For simplicity, *k* is assumed to be constant. The parameter 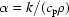 is called the thermal diffusion coefficient and determines the rate of the diffusion process. ρ_HS_ is the power density of the heat source derived from equation (2)
                  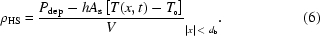
In order to solve equation (6) numerically, time and space were discretized. Potential stability problems were overcome by using the Crank–Nicolson method (Crank & Nicolson, 1996 ▶). Since the thin cryo-EM samples are relative transparent to the electron beam, heat diffusion in the direction of the beam (axial) can be considered instantaneous. As boundary conditions, it was assumed that the supporting copper mesh was in perfect thermal contact with the liquid-nitrogen-cooled sample-holder rod, and kept at a constant temperature of 103 K. The illuminated specimen area |*x*| < *d*_b_ (*d*_b_ being beam diameter) was approximated as a lumped system. Simulations were performed for vitreous ice of 50 µm diameter and 0.15 µm thickness, a uniform beam (a top-hat function) with a diameter of 10 µm, an incident flux density of both 5 e Å^−2^ s^−1^ and 50 e Å^−2^ s^−1^ at 120 kV accelerating voltage, and a heat-transfer coefficient *k* = 1.1 W m^−1^ K^−1^ (Kriminski *et al.*, 2003 ▶). Since the grid mesh is larger than the electron beam diameter, heat is transported from the illuminated region to the grid *via* the sample. Energy loss into the vacuum through black-body radiation has been neglected. The temperature difference between the grid and the edge of the illuminated specimen is given by 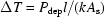, where 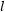 is the distance from illuminated area to the grid bars. If this is compared with the stationary case of the lumped system 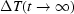 = 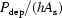, the heat-transfer coefficient *h* can be approximately expressed by 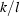.

#### 2.2.3. Mass loss

For each series the common subarea was defined and its mean intensity was calculated for each image. The slope of Δ*I*/*I*_0_ (Δ*I* = *I* − *I*_0_) *versus* dose was tabulated together with the intensity of the first image of each series, the estimated sample thickness, and the number of hemoglobin molecules per unit area.

#### 2.2.4. Beam-induced movement

Fiducial gold particles in the aligned images were used to track beam-induced movements that might have occurred during data collection. Distance matrices were calculated from the gold marker positions for the first and last image of each series. The movement of the gold particles was measured by a change in these distance matrices within a series. The mean of the distance differences provides a metric for beam-induced movements (Chen, Sachse *et al.*, 2008 ▶).

The gold marker detection was challenging because of several difficulties. The gold markers are on average 5 nm in diameter, but can vary significantly in shape and size. The different series showed differences in signal-to-noise ratio. Inspired by Lowe (2004 ▶) and Mikolajczyk *et al.* (2006 ▶) the above problems were overcome by using the Laplacian of Gaussian-filtered images. The Gaussian filtering was performed for a range of sigma values, varying around the gold size in pixels. The Laplacian of each of these Gaussian-filtered images were summed, which is defined here as the sum of the Laplacian of Gaussian functions (sLOG). Gold particles were detected as the brightest regions in the sLOG images. The centers of the gold particle positions were found from the center of mass of the brightest regions. For each gold particle in the reference image the vicinity of the area in the aligned image was used to locate the corresponding gold particle in that image.

#### 2.2.5. Figure-of-merit as a measure of phase error

After alignment, a common subarea was defined for each exposure series. The Fourier transforms (FT) of these subimages were averaged to yield averaged complex structure factors. A figure-of-merit was defined as
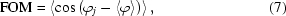
where ϕ*_j_* is the phase of the FT of individual subimage {*j*}, 〈ϕ〉 is the phase of the averaged complex structure factor described above, and the averaging is carried out for each pixel over *N* number of images within a series. *N* varied between 10 and 250 in our calculations. The FOM can vary between zero for random data and one for ideal noise-free data.

#### 2.2.6. Defocus estimation

Periodogram-averaged power spectra were calculated as described previously (Fernández *et al.*, 1997 ▶). The power spectra of the individual (medium- and low-dose) images were too noisy for defocus estimation through contrast transfer function (CTF) fitting.

The defocus could be derived from the radial averaging of the mean cosine of the difference phase, FOM (Karimi Nejadasl *et al.*, unpublished data). These FOMs were calculated after splitting each data series into five parts, with each part corresponding to an integrated flux density of 50, 100, 150, 200 and 250 e Å^−2^ respectively.

#### 2.2.7. Fourier ring correlation and Fourier ring phase residual

The radiation damage was scrutinized closely by different similarity metrics. Two metrics were computed, the Fourier ring correlation (FRC) and the Fourier ring phase residual (FRPR; Van Heel, 1987 ▶; Liao & Frank, 2010 ▶). They are obtained from
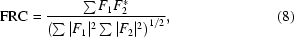

                  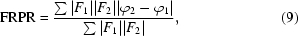
where 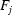, 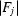 and 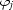 are, respectively, the Fourier transform of the *j*th image for 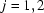 and its magnitude and phase. The metrics were computed up to the first crossing of the CTF, namely 166–3.5 nm. Images were first aligned and then summed up to the specified integrated flux density.

## 3. Results

### 3.1. Dose

Table 1 ▶ shows the relation between incident flux and dose for all the data. The dose was calculated based on the following parameters. The electron beam had a diameter of 10 µm as measured at lower magnification, using the same condenser and objective lens settings as for the experiments. Tomographic reconstructions (see §2.1.3) showed that the typical vitreous sample layer thickness was ∼150 nm. A volume of 11.8 fl was irradiated with, for the medium-flux exposure series, 5 e Å^−2^ s^−1^ during 1 s per image. The counted number of Hb molecules per unit area (for example 1000 molecules in 1 µm^2^) is given in Table 1 ▶. A density for low-density amorphous ice of 0.93 g cm^−3^ (Alcorn & Juers, 2010 ▶) was used, resulting in a total of 3.6 × 10^11^ water molecules in the path of the beam. Based on all these parameters, an approximation for the total atomic content of the irradiated volume could be calculated. The total mass of the irradiated volume, based on these atom counts, was 10.9 pg. Using the atomic scattering factors of Langmore & Smith (1992 ▶) and an incoming beam energy of 120 kV, we calculate that a fraction of 48% of the incoming electrons was scattered inelastically, each depositing 20 eV, resulting in a total amount of energy deposited of 60.1 nJ. The dose for each individual medium-flux image corresponds to ∼5.5 MGy.

### 3.2. Average intensity *versus* dose

**Figure 1:**
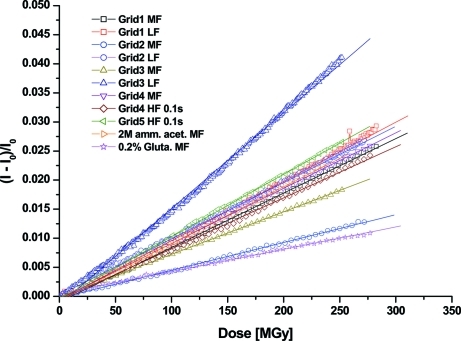
Normalized intensity change as a function of dose for different exposure series. LF refers to the low-flux series (1 e Å^−2^ s^−1^), MF to medium-flux (5 e Å^−2^ s^−1^) and HF to high-flux (50 e Å^−2^ s^−1^). The normalized intensity change was found to be linear with dose as shown by least-squares fit to the data.

Table 1 ▶ gives the slopes of the normalized intensity change Δ*I*/*I*_0_ *versus* dose for the common subareas of each exposure series. The different incident flux densities and integration times can be found in the same table, together with the dose (in gray) per exposure. The Δ*I*/*I*_0_ graphs are shown in Fig. 1 ▶: the metric is highly linear with dose for all the low-, medium- and high-flux short-exposure (0.1 s) series that were collected. However, the high-flux (1 s exposure) series had to be excluded due to non-uniform events such as gas bubble formation, image blurring or crystalline ice formation. The images became approximately 1% brighter per 100 MGy dose (0.1% per 10 MGy).

### 3.3. Radiation damage series of Hemoglobin followed up to 5500 MGy

Movie S1 (see supplementary materials^1^) shows a high-flux series of 100 images. Each image was taken with 50 e Å^−2^ s^−1^ and 1 s integration time, corresponding to a dose of ∼55 MGy per image. This series was taken at the edge of a hole of a C-flat support film, showing the support film on the right-hand side of the image. Comparing the first with the second image in this series, one can already observe a blurring of the particles. This loss of resolution proceeds monotonically throughout the first 10–20 images. Cryo-electron tomography regularly shows the presence of loose ice particles on top of the vitreous sample layer. In our movie, such ice particles can be seen in the lower-left part of the image. This ice crystal seems to dissolve into the sample layer within the first seven images. Starting from image number seven, macroscopic bubbles appear at the protein sites. This is most obvious for the vitreous sample layer in the hole. One to four nanobubbles appear per hemoglobin complex, and a maximum number of bubbles is seen around image number 14. Hereafter, bubbles fuse and, eventually, disappear. Most bubbles in the hole area have disappeared at image number 40. The structure of the individual protein complexes disintegrates together with the bubble formation. At image 10, a remnant of the sixfold symmetry can still be seen for some particles, whereas towards image 40 all resemblance with the original particles is gone. Strikingly, the relative positions of the fiducial gold markers do not seem to alter significantly. Later in the series, from frame 60 onwards, the images start to show more detail. Sharp black worm-like features form, residuals of the protein molecules. The whole series had to be corrected for a linear change in intensity, as the sample was becoming more transparent with dose (Fig. 1 ▶). After image number 97, a hole formed from the top part of the image. In total, an excessive dose of 5500 MGy was used for this series, which was collected over a time span of 7.5 min.

### 3.4. Defocus variation

Changes in image contrast and particle resolution could, in principle, be a consequence of a drift of the defocus during the exposure series acquisition. The general stability of the microscope, therefore, was investigated by imaging a thin layer of carbon at room temperature 30 times. A series of measurements at three consecutive levels of defocus was recorded: −1, −1.25 and −1.5 µm. The standard deviation of the series was in the range of a few nanometers (Vulovic, Brandt *et al.*, 2010 ▶).

**Figure 2:**
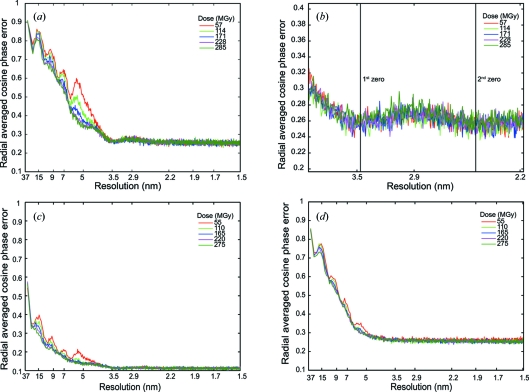
Plots of radial-averaged cosine phase error *versus* resolution for different dose rates. (*a*) Radial-averaged FOMs are given for a medium-flux series on Hb in a low-salt sample for integrated flux densities of 50, 100, 150, 200 and 250 e Å^−2^. (*b*) Close-up of (*a*) showing the first and second zero crossing of the CTF for a defocus of −3.37 µm. Radial-averaged FOMs for (*c*) the low-flux and (*d*) high-flux short-exposure series.

Radial averaged FOM figures were calculated [see equation (7)]. Fig. 2 ▶ shows these graphs for five different successive cumulative doses for a medium-flux series on Hb in a low-salt sample. The first and second zero of the CTF would correspond to 3.46 and 2.45 nm, respectively, for an estimated defocus of −3.57 µm (the requested defocus was −3 µm). Both positions are found in these data and do not drift significantly as a function of cumulative dose. Fig. 2 ▶ is representative in this respect of all of the exposure series used in this study. It is found that the defocus ranged between −2.83 and −3.57 µm for the different medium-flux series.

### 3.5. Beam-induced movement of gold particles

The mean value (and its standard deviation) of change in distance between all possible pairs of gold particles is shown in Table 1 ▶. The average values for all low, medium and high incident flux series data are found to be ∼2–8 pixels regardless of the solvent constituent used except for the glycerol sample. The value is about the same for the high-flux short-exposure series. A large distance of 54 pixels is seen between the first (integrated flux density 5 e Å^−2^) and fifth image (25 e Å^−2^) from the 50% glycerol medium-flux series. The value becomes 89 pixels when the first image is compared with the tenth image (50 e Å^−2^), indicating an excessive amount of beam-induced movements within the glycerol sample.

### 3.6. Fourier ring correlation, Fourier ring phase residual and FOM plots

**Figure 3:**
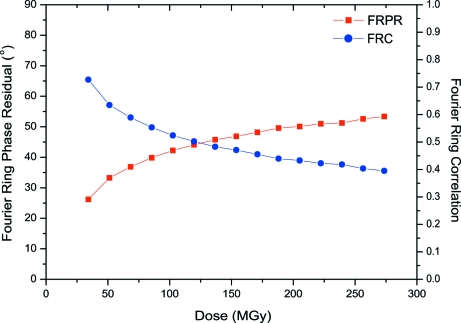
Fourier ring phase residual (FRPR) and Fourier ring correlation (FRC) as a function of dose. Medium-flux data were combined in groups of three images, corresponding to an integrated flux density of 15 e Å^−2^ per combined image. The first combined image was used as a reference.

The aligned medium-flux images were grouped over a variable number *N*. Fig. 3 ▶ shows FRC [see equation (8)] and FRPR [see equation (9)] for combined images that contain the sum of three original images. Each combined image corresponds to an integrated flux density of 15 e Å^−2^. The first summed image was taken as a reference and compared with the successive summed images within a series. The metrics were calculated for different resolution ranges: Fig. 3 ▶ shows only the low-resolution data. The FRC decreases as a function of cumulative dose, whereas the phase residual FRPR increases. Similarly, the FOM values decrease (corresponding to an increase in phase errors) as a function of accumulated dose for all three dose-rate series, low, medium and high flux (Figs. 2*a*, 2*c* and 2*d* ▶).

### 3.7. Dose-rate effects

**Figure 4:**
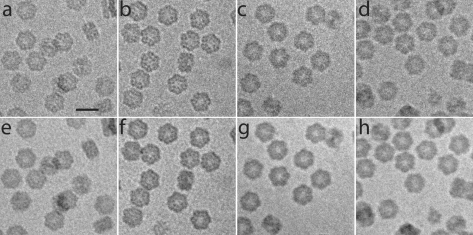
Qualitative investigation of the dose-rate effect. The aligned and summed images of (*a*) and (*e*) low-flux, (*b*) and (*f*) medium-flux, (*c*) and (*g*) high-flux, and (*d*) and (*h*) high-flux short-exposure series are shown at two different integrated flux densities of (*a*)–(*d*) 50 e Å^−2^ and (*e*)–(*h*) 250 e Å^−2^, respectively. The scale bar shown in (*a*) corresponds to 30 nm.

Fig. 4 ▶ shows the summed image of an aligned low-flux (Fig. 4 ▶ *a*), medium-flux (Fig. 4 ▶ *b*) and high-flux short-exposure (Fig. 4 ▶ *d*) series of a control set of Hb for an equal integrated flux density of 50 e Å^−2^. As a comparison, the first image of a high-flux series (50 e Å^−2^ s^−1^, 1 s exposure) collected from the same grid is also shown (Fig. 4 ▶ *c*). Figs. 4 ▶(*e*)–4(*h*) show respective images for an integrated flux density of 250 e Å^−2^. It can be seen that for an equal accumulated incident flux, the images of the high-flux series are invariably blurred. Furthermore, for an equal integrated flux density, the appearance of gas bubbles (data not shown) occurred earlier in the high-flux short-exposure series compared with the medium- and low-flux series.

Figs. 2 ▶(*c*) and 2(*d*) show FOM plots for the low-flux and high-flux short-exposure series, respectively. The identical dose was fractionated over the same number of images as plotted in Figs. 2 ▶(*a*) and 2(*d*). Both graphs start with comparable FOMs at low resolution, but fewer high-resolution details can be seen for the high-flux short-exposure series. The low-flux series (Fig. 2 ▶ *c*) shows high-resolution details, although in absolute terms all FOMs are smaller compared with the medium-flux series, probably due to an accumulation of alignment errors for the fivefold larger number of images. We measured camera statistics such as readout noise and dark current (Vulovic, Rieger *et al.*, 2010 ▶) and note that these sources of error are relatively small even for the low-flux series.

### 3.8. Heating effects

**Figure 5:**
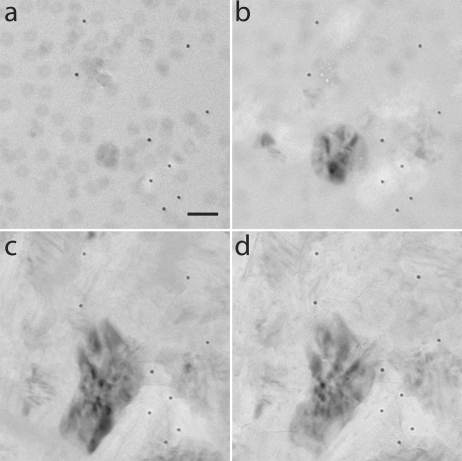
Beam-induced ice crystallization at a high-flux rate (50 e Å^−2^ s^−1^). Images (*a*)–(*d*) were all taken in the same series and correspond to doses of 57, 565, 1695 and 2825 MGy, respectively. The beam-induced movement calculated for the fiducial gold markers in these images is surprisingly small (see Table 1 ▶, grid 1, high-flux series). The scale bar shown in (*a*) corresponds to 60 nm.

Electron irradiation could induce ice crystallization in the sample, as observed for the high-flux exposure series on the 50 m*M* NH_4_Ac sample (Fig. 5 ▶). In another high-flux series, during which 100 images were collected, ice crystallization was not observed: instead, dark flake-like particles appeared prior to a complete sublimation of the illuminated area at a cumulative dose of 5500 MGy (Movie S1). Could this crystallization be due to sample heating?

**Figure 6:**
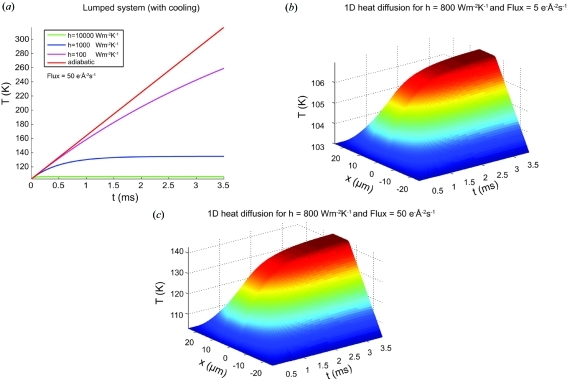
Simulations of the heating of a vitreous sample by the electron beam. (*a*) Temperature *versus* time plot for a lumped system model for three different heat-transfer coefficients *h*. The adiabatic model temperature rise is shown in comparison. One-dimensional heat-diffusion plots for a distributed model are shown for (*b*) medium- and (*c*) high-flux incident beam for a low heat-transfer coefficient of 800 W m^−2^ K^−1^.

The calculated dose for the parameters given in §3.1 is 5.5 MGy. Heating simulations for a sample treated as a lumped system are shown in Fig. 6 ▶(*a*) for different values of *h* and compared with those for an adiabatic process. Figs. 6 ▶(*b*) and 6(*c*) show the temperature distribution calculated for the distributed model [see equation (5)] for the medium- (5.5 MGy s^−1^) and high-flux series (55 MGy s^−1^). The simulated temperature rise is strongly dependent on the incident flux and on the cooling rate given by the heat-transfer coefficient *h*. For *h* = 800 W m^−2^ K^−1^, the temperature is predicted to rise within milliseconds from 103 to 140 K when using the high-flux density of 50 e Å^−2^ s^−1^.

### 3.9. The role of solvent constituents

**Figure 7:**
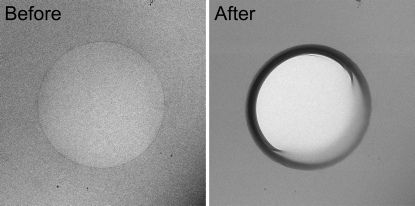
Low-magnification image of Hb sample in 50% (*v*/*v*) glycerol before and after the collection of a medium-flux series data. The hole (sized 1.2 µm) in the carbon support film contains a thin layer of vitreous sample in the before image, which is completely destroyed after the collection of 50 images, corresponding to a dose of 270 MGy.

**Figure 8:**
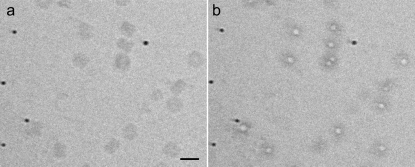
Representative high-flux images from (*a*) the first and (*b*) the 12th exposure from a 0.2% glutaraldehyde sample. Clear bubbling can be observed on every protein particle. The scale bar shown in (*a*) corresponds to 30 nm.

Four different solvent constituents were used: 50 m*M* NH_4_Ac, 2 *M* NH_4_Ac, 50% (*v*/*v*) glycerol and 0.2% (*v*/*v*) glutaraldehyde. Among these, the images of the higher-density glycerol sample (Fig. 7 ▶) showed less contrast compared with other samples, although the requested defocus was the same for all the exposure series collected. The beam-induced movements were excessive for the medium-flux series of the glycerol sample: these movements occurred concurrently with the formation of gas bubbles. At high-flux, gas bubbles formed in all the samples. Among the solvents studied, gas bubble formation within the high-flux series was most clearly localized at the protein sites for the 0.2% glutaraldehyde sample (Fig. 8 ▶).

## 4. Discussion

### 4.1. Dose

The incident flux density is a poor metric to use for radiation damage studies, as the probability of the sample–electron interaction does, apart from the incident flux density, depend on the integration time, the sample and the electron energy. Whereas an older paper on specimen damage (Stenn & Bahr, 1970 ▶) calculates the absorbed dose in energy per mass unit (erg g^−1^, 1 erg = 10^−7^ J), most recent electron microscopy papers use e Å^−2^ as the unit for dose. Analogous to dose calculations carried out for MX (Murray *et al.*, 2004 ▶), we estimated the absorbed dose in gray based on the electron-beam energy and size, the protein concentration, sample thickness, incident flux, exposure time and tabulated inelastic scattering coefficients.

The typical integrated flux densities used in SP cryo-EM range between 15 and 25 e Å^−2^ (Frank, 2009 ▶). For example, Zhang *et al.* (2010 ▶) recorded micrographs at approximately 25 e Å^−2^ for the 3.3 Å reconstruction of a primed aquareovirus. Cope *et al.* (2010 ▶) took single frame images at 15 e Å^−2^ for the study of kinesin-microtubule complexes, whereas Chen *et al.* (2008 ▶) used 25–36 e Å^−2^ for bacteriorhodopsin and ∊ 15 bacteriophage. The typical integration time is 1 s, although the latter authors used 1.4 and 2 s. For helical reconstruction or cryo-electron tomography studies, a larger integrated flux density is used, corresponding to 40–100 e Å^−2^ (Cope *et al.*, 2010 ▶; Baker & Rubinstein, 2010 ▶) or even 24–150 e Å^−2^ (Bárcena & Koster, 2009 ▶). In tomography, the dose is divided over a large number of images (McEwen *et al.*, 1995 ▶).

Table 1 ▶ shows the relation between incident flux density, integration time and dose for the data presented here. The dose used to record the individual images of the medium-flux series with an incident flux density of 5 e Å^−2^ s^−1^ varies between 5 and 6 MGy. For our sample and the electron energy used, the ‘Henderson’ dose limit (20 MGy: Henderson, 1990 ▶) and the ‘Garman’ dose limit (30 MGy: Owen *et al.*, 2006 ▶) would correspond to an integrated flux density of ∼20 and ∼30 e Å^−2^, respectively. Unlike MX, cryo-EM offers a unique way to study the decay of macromolecules at doses that exceed these limits by at least one order of magnitude (Movie S1).

Fig. 3 ▶ illustrates the gradual alteration of the low-resolution information in our data. Here, the medium-flux data are combined in groups of three images, corresponding to an integrated flux density of 15 e Å^−2^ per combined image. According to the criteria of the FRPR function being less than 45° and the FRC value being larger than 0.5 (Van Heel, 1987 ▶; Liao & Frank, 2010 ▶), one could combine these low-resolution data up to 125 MGy. However, these criteria would indicate that one could also combine data from, for example, 50–150 MGy yielding good statistics on radiation-damage compromised particles. The main cause of loss of correlation is the spread in radiation-damage-induced particle conformations.

Equation (1) gives an empirical relation between radiation damage, dose and resolution obtained from MX studies. According to this formula, the same fractional loss of diffracted intensities is obtained for constant ratios of dose over resolution length. Thus radiation damage observations obtained with high dose at low resolution would also be of relevance for lower dose at high resolution. Cryo-electron tomography is, compared with SP cryo-EM, a lower resolution technique that is performed with a higher dose. We hypothesize that a relation similar to (1) exists for SP cryo-EM: the rate of loss of signal at high dose at low resolution is likely to be related to the rate of loss of signal with low dose at high resolution.

We would advocate the collection of data series (‘stroboscopic data collection’; Typke *et al.*, 2007 ▶) rather than individual images, with a dose ranging from, for example, 5 to 100 MGy. For particle picking, radiation damage is less of a problem. In fact, the gas bubble formation observed at a higher dose could even be helpful in locating the particles (Fig. 8 ▶). Radiation-damage compromised images might still be useful for alignment, as a minimum dose is required to align particles of a certain size to a certain resolution (Saxton & Frank, 1977 ▶; Frank, 2009 ▶). Constant-dose interpolation schemes could be explored for stroboscopic data, similar to that which has been implemented in MX (Diederichs *et al.*, 2003 ▶). Alternatively, only the very first few images from each dose series could be used in the final reconstruction steps, providing a minimal and controlled amount of damage.

Radiation damage should not be treated as a binary nuisance, neither in MX nor in SP cryo-EM. Right from the first exposure of the sample to ionizing radiation, structural changes will occur (Burmeister, 2000 ▶; Ravelli & McSweeney, 2000 ▶; Weik *et al.*, 2000 ▶). The amount of dose is the main determinant of the amount of radiation damage.

### 4.2. Dose-rate effect

We observed a dose-rate effect in accordance with Chen *et al.* (2008 ▶) who introduced a LINDA imaging protocol: Low Intensity aNd low Dose Acquisition. For the same integrated flux density of 15 e Å^−2^, Chen *et al.* (2008 ▶) compared data that were collected with an incident flux density of 15 e Å^−2^ s^−1^ for 1 s (HiFlux) with data collected at 1.5 e Å^−2^ s^−1^ for 10 s (LINDA). Reconstructed models from successive data sets showed fewer signs of radiation damage for the data that were collected with the LINDA protocol compared with the HiFlux data. The 10 s data collection poses strict requirements on the cryo-stage of the electron microscope, as the sample should move less than a fraction of a pixel (*e.g.* <1 Å) within that time. Chen *et al.* used a FEI Polara microscope, whereas our analyses were based on data that were collected with a more common electron microscope, a FEI Tecnai T12. The mechanical drifting of the stage was overcome by dose fractionation. Fig. 4 ▶(*b*) shows a summed image for the medium-flux data, where ten images of 5 e Å^−2^ s^−1^ with 1 s integration time were aligned and added. The low-flux series (Fig. 4 ▶ *a*), where 50 images of 1 e Å^−2^ s^−1^ with 1 s integration time were added, showed less detail, possibly because of the accumulation of alignment errors due to the low signal-to-noise ratios in the individual images. Fig. 4 ▶(*c*) comes from a single image, taken at 50 e Å^−2^ s^−1^ with a 1 s integration time. This figure is representative of all high-flux series which never showed great detail. Adding ten aligned high-flux images (50 e Å^−2^ s^−1^) recorded with short exposure times (0.1 s) did not show clear improvements. Figs. 4 ▶(*e*)–4(*h*) show corresponding images for an integrated flux density of 250 e Å^−2^.

A more quantitative analysis of these images is shown in Fig. 2 ▶. We introduced a new metric, analogous to MX, for ascertaining phase qualities, namely the average cosine of phase errors (FOM). The FOM plots enabled us to estimate the defocus values from the images taken from the vitreous sample area that excludes any carbon support (Fig. 2 ▶ *b*).

The medium-flux series (Fig. 2 ▶ *a*) shows the most detail at higher resolution compared with the high-flux (Fig. 2*d* ▶) and low-flux (Fig. 2*c* ▶) series. Unlike the high-flux series, there is still a signal between the first and second zero of the CTF (Fig. 2*b* ▶) in the low-flux series (Fig. 2*c* ▶). We believe that this signal has been dampened due to an accumulation of alignment errors for the larger number of images used in the low-flux series, a problem that would be overcome by the LINDA protocol. Alternatively, the use of larger fiducial markers combined with more sophisticated alignment schemes could help when the data are fractioned over a larger number of images.

Fig. 2(*a*) ▶ shows the medium-flux series, grouped in subsets of ten images corresponding to an integrated flux density of 50 e Å^−2^. The peaks observed in this graph relate to the radial averaged Fourier transform of the hemoglobin particles. They are most pronounced for the medium-flux series (Fig. 2*a* ▶), demonstrating that this series not only provides higher-resolution data but also a better signal at lower resolution. The peaks quickly reduce in height as a function of dose: at higher resolution the loss of signal is faster than at lower resolution.

Chen *et al.* (2008 ▶) observed larger beam-induced movements of the fiducial markers for the high-flux series. This effect is not obvious from our data (Table 1 ▶) as the beam-induced movements, measured between the first and last image of each series and given in pixels, are scattered. A typical value is 5 pixels, although the range is between 1 and 9 pixels. Chen *et al.* used 10 nm gold particles and frames recorded with 15 e Å^−2^, whereas we used 5 nm gold particles and frames recorded with 1, 5 or 50 e Å^−2^. The uncertainty in fiducial marker localization is larger for most of our data. The fiducial marker distances between successive images varied between two pixels for the low-dose series and one pixel for the high-flux series. For some of the data series, part of the beam-induced movements could be modelled with global parameters such as scaling or rotation. It is remarkable how little the fiducial markers move in the extreme case of ice crystallization (Fig. 5 ▶), challenging the credence of using differences in gold position distances as a metric for beam-induced movements.

Analogous to MX studies (Ravelli *et al.*, 2002 ▶; Southworth-Davies *et al.*, 2007 ▶), we discuss two possible causes for the observed dose-rate effect: excessive heating and radical recombination.

#### 4.2.1. Sample heating

Sample heating could cause dose-rate effects, since the balance between heating by the electron beam and cooling by conduction will depend on the rate the energy is deposited in the sample. Analogous to Kuzay *et al.* (2001 ▶) and Kriminski *et al.* (2003 ▶), we simulated the heating of the vitrified sample using a lumped and a distributed model for different values of the heat-transfer coefficient *h*. In the adiabatic case a thermally isolated sample of the same size as the beam would melt quickly (Fig. 6*a* ▶). Both the lumped and the distributed models indicate that the temperature will rise most rapidly within the first milliseconds after exposure of the sample to the electron beam. Compared with MX, the system time constant [see equation (4)] is much smaller in SP cryo-EM due to the lower volume–surface ratio and the larger heat-transfer coefficient. Figs. 4(*d*) and 4(*h*) ▶ seem to indicate that fast (sub-100 ms) processes are indeed responsible for the observed dose-rate effects. The images from the series of Figs. 4(*b*) and 4(*d*) ▶ were recorded with the same integrated flux density per image, namely 5 e Å^−2^; however, the images from Fig. 4 ▶(*b*) were integrated over 1 s at 5 e Å^−2^ s^−1^ whereas the images from Fig. 4 ▶(*d*) were integrated over 0.1 s at 50 e Å^−2^ s^−1^. The latter images are clearly worse, indicating that the additional damage induced by the high flux occurs in less than 100 ms.

Only for very high dose rates and low values of *h*, representing, for example, poor thermal contact between the grid and the cryo-holder, is sample heating predicted to become an issue for SP cryo-EM, as the temperature of the sample is calculated to rise (Fig. 6 ▶ *c*) above the glass transition (Weik *et al.*, 2001 ▶; Weik & Colletier, 2010 ▶), triggering an exothermic ice crystallization process. In fact, for one high-flux series, radiation-induced ice crystallization was observed (Fig. 5 ▶). However, this result was exceptional, suggesting poor thermal contact for that particular grid.

The heat model presented here complements existing specimen heating models as used in TEM (see, for example, Reimer & Kohl, 2008 ▶) and could form the basis for an elaborate refinement that studies the influence of supporting mesh size, size and spacing of holes within the support film, distance of the beam with respect to the grid bars, *etc*. Some experimental verification of *h* for different combinations of grids and holders would be required (Reimer & Kohl, 2008 ▶). Such studies are beyond the scope of this manuscript; however, we can postulate that the effect of beam heating is felt within milliseconds after exposure, and beam heating is not expected to be a problem for cryo-EM samples with good thermal contact at medium- or low-flux densities.

#### 4.2.2. Radical recombination

In MX it is believed that the photo-electric absorption of a ∼12 keV X-ray photon will produce ∼500 radicals, assuming 25 eV per ionization event (O’Neill *et al.*, 2002 ▶). For our medium-flux data series taken at 5 e Å^−2^, we estimated 1.9 × 10^10^ inelastic scattering events per frame within a volume of 11.8 fl. If each inelastic event acts on a different target and produces one radical and ignoring radical recombination processes, then the radical concentration at the end of the first exposure would be 2.6 *M*. We extrapolate that for typical SP cryo-EM data collections the biological molecules would be exposed to molar concentrations of radicals. Some of these radicals, in particular electrons, must be mobile (Ravelli & McSweeney, 2000 ▶) as the damage seems to accumulate at the interface of protein sites (see Movie S1; Fig. 8 ▶; Glaeser *et al.*, 2007 ▶; Baker *et al.*, 2010 ▶).

Ignoring radiation recombination processes, one would calculate 52 *M* as the radical concentration for the high-flux series after 2 s of exposure, which is comparable with the concentration of water within the sample. Such radical concentrations are unlikely to be present, thus radical recombination must play a role for our data.

Dose-rate effects could be caused by concentration-dependent radical chemistry and the diffusion of gas molecules within the sample. Supplementary Movie S1 illustrates the formation, diffusion, fusion and rupture of these bubbles. For high-intensity beams, the pressure can become so high that it generates mechanical fractures within the specimen (Chen, Sachse *et al.*, 2008 ▶), and since this would negatively effect the conductive cooling of the sample it might lead to local beam heating. The absolute temperature of the sample could play a role for dose and dose-rate effects: recently, a temperature of 50 K instead of 100 K was shown to reduce specific damage in MX by a factor of three to four (Meents *et al.*, 2010 ▶), whereas, for cryo-EM diffraction studies, 100 K was found to be the optimal temperature (Bammes *et al.*, 2010 ▶). Higher dose rates could also lead to an inverse dose-rate effect, as radical recombination could become more important, in particular at elevated temperatures (Southworth-Davies *et al.*, 2007 ▶). The dose rates used in this SP cryo-EM study varied between 1 and 56 MGy s^−1^, which is very high compared with the dose-rate studies carried out in MX [*e.g.* Southworth-Davies *et al.* (2007 ▶) used 6–10 Gy s^−1^; Cherezov *et al.* (2002 ▶) used 20–6400 Gy s^−1^; Leiros *et al.* (2006 ▶) used 0.2 MGy s^−1^]. The data recorded with 56 MGy s^−1^ were inferior to the lower dose-rate series. This raises the question as to whether the typical dose rate used in SP cryo-EM (∼25 MGy s^−1^) is optimal. It would be worth investigating whether further improvements could be obtained by lowering the dose rate in SP cryo-EM studies by another order of magnitude. Simulations suggest that it should be possible to align extremely low-dose images for essentially noise- and point-spread function-free detectors (Typke *et al.*, 2007 ▶). Actual developments in detector technology yield promise for dose fractioning in SP cryo-EM.

The high dose rates used in SP cryo-EM make it likely that radiation chemistry will play an even larger role compared with MX. There are indications that scavengers could prolong the lifetime of cryo-cooled crystals in the X-ray beam (Kauffmann *et al.*, 2006 ▶; Southworth-Davies & Garman, 2007 ▶; De la Mora *et al.*, 2011 ▶) by neutralizing immobile ionized groups or quenching radical species. Unfortunately, the addition of a high concentration of scavengers can be harmful for fragile protein crystals. This difficulty does not exist in SP cryo-EM, although other problems, such as reduced sample contrast, might arise.

Hydrogen trapping was proposed (Meents *et al.*, 2010 ▶) to be the cause of unit-cell volume expansion observed in MX (Ravelli *et al.*, 2002 ▶). In SP cryo-EM the sample shrinks with dose, as radiolytic products, in particular hydrogen gas, diffuse out of the sample into the high-vacuum column of the electron microscope, resulting in mass loss. This process is linear with dose and seems to be highly reproducible among different samples tested (Fig. 1 ▶). The observed linear relationship between the relative intensity change and the dose could be a useful metric for studying the effects of scavengers.

Other metrics presented in this manuscript include FOM (Fig. 2 ▶), Fourier ring correlation and Fourier ring phase residual (Fig. 3 ▶), and beam-induced movements (Table 1 ▶). Here, radioprotectants were not tested but rather one fixative and two cryoprotectants, among which was glycerol, the most widely used cryoprotectant in MX. The 50% glycerol sample showed very little contrast between the protein and the solvent as its density (1.181 g cm^−3^ at 72 K; Alcorn & Juers, 2010 ▶) is comparable with the average density of protein molecules (1.35 g cm^−3^). Bubbling was observed throughout the glycerol sample, not only at the protein sites, consistent with the discussion by Meents *et al.* (2010 ▶) that hydrogen gas (Leapman & Sun, 1995 ▶) is formed upon radiolysis of organic molecules. Within 50 medium-flux images, the vitrified layer of the sample within the hole was completely sublimated (Fig. 7 ▶), unlike the other samples at medium-flux (Figs. 4*e*–4*h* ▶). The gold fiducial markers showed large beam-induced movements (Table 1 ▶). The observed increased sensitivity to radiation damage upon addition of glycerol calls for further studies, in particular for MX.

The 2 *M* NH_4_Ac sample did not show clear differences in radiation damage susceptibility: the relative intensity change (Fig. 1 ▶) and beam-induced movements (Table 1 ▶) were comparable with the low-salt samples. The distribution of the Hb particles within the sample was slightly different, as some Hb particles packed regularly. Similar to all the other samples, the 2 *M* NH_4_Ac sample was vitrified in liquid ethane. High concentrations of salt are routinely used as cryoprotectants in MX: we could have vitrified this sample with liquid nitrogen, thus overcoming some of the disadvantages of using liquid ethane.

The localized appearance of gas bubbles was most obvious for the 0.2% glutaraldehyde sample (Fig. 8 ▶). Kastner *et al.* (2008 ▶) advocated the use of 0.2% glutaraldehyde for improving the sample quality for structure determination by SP cryo-EM. The described benefits of using a chemical fixation reagent in stabilizing individual macromolecules during sample preparation might also help in keeping the macromolecules together upon radiolysis.

## 5. Conclusions

Radiation damage should not be treated as a binary nuisance. It gradually changes the quality of SP cryo-EM data: the amount of alteration that is acceptable depends on what one aims for, for example, for particle picking or defocus estimation, a larger dose could be used compared with the calculation of a three-dimensional reconstruction. We advocate the use of stroboscopic data collection, with which variable amounts of dose can be used for the different steps of SP reconstruction.

Throughout this paper the gray is used as the unit of dose. It is estimated from the incident flux density, beam size, sample composition and thickness, and beam energy. The use of this unit provides direct access to the power deposited in the sample, which has been used for beam heating simulations. Furthermore, it allowed us to make direct comparisons with systematic radiation damage studies in MX, yielding, among other parameters, an upper estimate of the radical concentrations formed during cryo-EM experiments.

The usual dose applied in SP cryo-EM to collect single images is similar to the experimental dose limit for MX (30 MGy; Owen *et al.*, 2006 ▶) that is typically used to collect an entire data set of hundreds of diffraction images. These high doses in SP cryo-EM are necessary to counteract the low signal-to-noise ratios, but will inevitably cause radiation damage issues. The use of dose (in gray) is expected to be of help in characterizing the exact extent of these issues now that higher-resolution SP cryo-EM studies are more frequently being performed. Unlike MX, SP cryo-EM could offer a unique insight into the later stages of radiation damage to macromolecules, as one could continue to record SP cryo-EM data at doses that exceed 30 MGy by at least one order of magnitude.

A clear dose-rate effect could be observed, favoring lower flux rates. Data that were collected with an incident flux density of 50 e Å^−2^ s^−1^ were inferior in quality to those that were collected at 5 e Å^−2^ s^−1^. Beam heating simulations indicate that:

(i) the effect of beam heating is felt within milliseconds after exposure, and

(ii) beam heating is not expected to be a problem for cryo-EM samples with good thermal contact at medium- or low-flux densities.

The electron beam deposits enough energy to form molar concentrations of radicals and radical recombination is likely to play a role in the observed dose-rate effects. This gives hope for future scavenger studies. A number of metrics have been presented, such as relative intensity change *versus* dose, FOM, FRC, FRPR and beam-induced movements, which could aid such studies.

## Supplementary Material

Supplementary material file. DOI: 10.1107/S090904951100820X/xh5022sup1.avi

## Figures and Tables

**Table 1:** Mass loss upon electron-beam irradiation

Solvent, estimated number of Hb molecules (Hb µm^−2^), sample thickness (nm)	Incident flux density (e Å^−2^ s^−1^), integration time (s), dose per exposure (MGy)	*I*_0_ (ADU)	Δ*I*/*I*_0_*versus* dose (10^−10^ Gy^−1^)†	Beam-induced movements [pixels (std)]‡
50 m*M* NH_4_Ac, 500, 140	5, 1, 5.7	1420	0.915	4.2 (3.0)
		1457	0.937	2.1 (1.6)
		1240	0.921	4.9 (3.7)
	1, 1, 1.1	306	1.02	3.6 (2.7)
		307	0.949	2.8 (2.1)
		298	1.03	5.3 (4.4)
	50, 1, 56.5	18543		0.9 (0.8)
50 m*M* NH_4_Ac, 520, 160	5, 1, 5.4	1056	0.515	2.3 (1.8)
		1028	0.479	5.5 (3.0)
		1027	0.453	5.2 (3.5)
		998	0.456	5.5 (3.3)
	1, 1, 1.1	281	0.968	2.8 (2.0)
		289	0.966	3.1 (3.2)
		281	0.969	2.6 (1.9)
50 m*M* NH_4_Ac, 700, 200	5, 1, 5.0	1234	0.719	2.8 (2.1)
		1134	0.735	2.9 (2.1)
		1184	0.700	5.0 (3.4)
	1, 1, 1.0	406	1.97	8.8 (5.4)
		429	1.65	8.8 (5.4)
		428	1.63	7.3 (4.4)
50 m*M* NH_4_Ac, 570, 150	5, 1, 5.5	2083	0.898	2.0 (1.8)
		2025	0.887	2.9 (2.1)
		2075	1.07	3.7 (2.8)
		2132	0.953	2.4 (2.0)
		2115	0.978	2.7 (1.8)
	50, 0.1, 5.5	1996	0.862	6.9 (4.9)
		2009	0.707	5.6 (4.5)
		1886	0.905	2.5 (2.2)
		1910	0.860	3.4 (2.3)
		1987	0.878	2.7 (2.0)
	50, 1, 55.4	19359		0.9 (0.7)
50 m*M* NH_4_Ac, 690, 200	50, 0.1, 5.0	1422	0.956	3.3 (2.7)
		1403	0.874	3.0 (3.0)
		1369	1.11	1.9 (1.6)
		1304	1.10	2.1 (1.4)
		1396	1.07	4.2 (4.0)
2 *M* NH_4_Ac, 1120, 240	5, 1, 4.7	1494	1.03	5.0 (4.0)
		1517	0.973	
		1452	0.928	
		1469	0.976	
		1458	0.942	
0.2% glutaraldehyde, 120, 150	5, 1, 5.5	1547	0.217	8.2 (6.1)
		1417	0.384	
		1593	0.291	
50% glycerol,§ 280 , 150	5, 1, 5.4	1566		53.9, 89.5¶

† The correlation coefficients for all linear fits are around 0.99.

‡ First and last images within each series were compared.

§ The glycerol sample showed large movements of the fiducial gold markers.

¶ The two values correspond to the first to the fifth, and the first to the tenth image of the series, respectively.

## Footnotes

1 Supplementary data for this paper are available from the IUCr electronic archives (Reference: XH5022). Services for accessing these data are described at the back of the journal.

## References

[bb1] Alcorn, T. & Juers, D. H. (2010). *Acta Cryst.* D**66**, 366–373. 10.1107/S090744490903995X PMC2852300 20382989

[bb2] Baker, L. A. & Rubinstein, J. L. (2010). *Methods Enzymol.* **481**, 371–388. 10.1016/S0076-6879(10)81015-8 20887865

[bb3] Baker, M. L., Zhang, J., Ludtke, S. J. & Chiu, W. (2010). *Nat. Protoc.* **5**, 1697–1708. 10.1038/nprot.2010.126 PMC3107675 20885381

[bb4] Bammes, B. E., Jakana, J., Schmid, M. F. & Chiu, W. (2010). *J. Struct. Biol.* **169**, 331–341. 10.1016/j.jsb.2009.11.001 PMC2826528 19903530

[bb6] Bárcena, M. & Koster, A. J. (2009). *Semin. Cell Dev. Biol.* **20**, 920–930. 10.1016/j.semcdb.2009.07.008 PMC7110493 19664718

[bb7] Barker, A. I., Southworth-Davies, R. J., Paithankar, K. S., Carmichael, I. & Garman, E. F. (2009). *J. Synchrotron Rad.* **16**, 205–216. 10.1107/S0909049509003343 19240332

[bb8] Bhushan, S., Gartmann, M., Halic, M., Armache, J. P., Jarasch, A., Mielke, T., Berninghausen, O., Wilson, D. N. & Beckmann, R. (2010). *Nat. Struct. Mol. Biol.* **17**, 313–317. 10.1038/nsmb.1756 20139981

[bb9] Burmeister, W. P. (2000). *Acta Cryst.* D**56**, 328–341. 10.1107/s0907444999016261 10713520

[bb10] Carpentier, P., Royant, A., Ohana, J. & Bourgeois, D. (2007). *J. Appl. Cryst.* **40**, 1113–1122.

[bb11] Chen, D. H., Jakana, J., Liu, X., Schmid, M. F. & Chiu, W. (2008). *J. Struct. Biol.* **163**, 45–52. 10.1016/j.jsb.2008.04.001 PMC2504495 18514542

[bb12] Chen, J. Z., Sachse, C., Xu, C., Mielke, T., Spahn, C. M. & Grigorieff, N. (2008). *J. Struct. Biol.* **161**, 92–100. 10.1016/j.jsb.2007.09.017 PMC2213720 17977018

[bb13] Cherezov, V., Riedl, K. M. & Caffrey, M. (2002). *J. Synchrotron Rad.* **9**, 333–341. 10.1107/s0909049502014528 12409619

[bb14] Cope, J., Gilbert, S., Rayment, I., Mastronarde, D. & Hoenger, A. (2010). *J. Struct. Biol.* **170**, 257–265. 10.1016/j.jsb.2009.12.004 PMC2856765 20025975

[bb15] Crank, J. & Nicolson, P. (1996). *Adv. Comput. Math.* **6**, 207–226.

[bb16] De la Mora, E., Carmichael, I. & Garman, E. F. (2011). *J. Synchrotron Rad.* **18**, 398–412. 10.1107/S0909049511007163 21525642

[bb17] Diederichs, K., McSweeney, S. & Ravelli, R. B. G. (2003). *Acta Cryst.* D**59**, 903–909. 10.1107/s0907444903006516 12777808

[bb18] Fernández, J.-J., Sanjurjo, J. & Carazo, J.-M. (1997). *Ultramicroscopy*, **68**, 267–295.

[bb19] Frank, J. (2009). *Q. Rev. Biophys.* **42**, 139–158. 10.1017/S0033583509990059 PMC2844734 20025794

[bb20] Fujii, T., Iwane, A. H., Yanagida, T. & Namba, K. (2010). *Nature (London)*, **467**, 724–728. 10.1038/nature09372 20844487

[bb22] Garman, E. F. (2010). *Acta Cryst.* D**66**, 339–351. 10.1107/S0907444910008656 PMC2852297 20382986

[bb23] Glaeser, R. M. (2008). *J. Struct. Biol.* **163**, 271–276. 10.1016/j.jsb.2008.06.001 18588985

[bb24] Glaeser, R. M., Downing, K., DeRosier, D., Chiu, W. & Frank, J. (2007). *Electron Crystallography of Biological Macromolecules.* New York: Oxford University Press.

[bb25] Henderson, R. (1990). *Proc. R. Soc. London Ser. B*, **241**, 6–8.

[bb26] Henderson, R. (1995). *Q. Rev. Biophys.* **28**, 171–193. 10.1017/s003358350000305x 7568675

[bb27] Holton, J. M. (2009). *J. Synchrotron Rad.* **16**, 133–142.

[bb28] Holton, J. M. & Frankel, K. A. (2010). *Acta Cryst.* D**66**, 393–408. 10.1107/S0907444910007262 PMC2852304 20382993

[bb29] Howells, M. R., Beetz, T., Chapman, H. N., Cui, C., Holton, J. M., Jacobsen, C. J., Kirz, J., Lima, E., Marchesini, S., Miao, H., Sayre, D., Shapiro, D. A., Spence, J. C. & Starodub, D. (2009). *J. Electron Spectrosc. Relat. Phenom.* **170**, 4–12. 10.1016/j.elspec.2008.10.008 PMC2867487 20463854

[bb30] Iancu, C. V., Wright, E. R., Heymann, J. B. & Jensen, G. J. (2006). *J. Struct. Biol.* **153**, 231–240. 10.1016/j.jsb.2005.12.004 16427786

[bb31] Jones, G. D., Lea, J. S., Symons, M. C. & Taiwo, F. A. (1987). *Nature (London)*, **330**, 772–773. 10.1038/330772a0 2827033

[bb32] Jonic, S. & Vénien-Bryan, C. (2009). *Curr. Opin. Pharmacol.* **9**, 636–642. 10.1016/j.coph.2009.04.006 19464952

[bb33] Kastner, B. *et al.* (2008). *Nat. Methods*, **5**, 53–55. 10.1038/nmeth1139 18157137

[bb34] Kauffmann, B., Weiss, M. S., Lamzin, V. S. & Schmidt, A. (2006). *Structure*, **14**, 1099–1105. 10.1016/j.str.2006.05.015 16843891

[bb35] Kmetko, J., Husseini, N. S., Naides, M., Kalinin, Y. & Thorne, R. E. (2006). *Acta Cryst.* D**62**, 1030–1038. 10.1107/S0907444906023869 16929104

[bb36] Kremer, J. R., Mastronarde, D. N. & McIntosh, J. R. (1996). *J. Struct. Biol.* **116**, 71–76. 10.1006/jsbi.1996.0013 8742726

[bb37] Kriminski, S., Kazmierczak, M. & Thorne, R. E. (2003). *Acta Cryst.* D**59**, 697–708. 10.1107/s0907444903002713 12657789

[bb38] Kuzay, T. M., Kazmierczak, M. & Hsieh, B. J. (2001). *Acta Cryst.* D**57**, 69–81. 10.1107/s0907444900013299 11134929

[bb39] Langmore, J. P. & Smith, M. F. (1992). *Ultramicroscopy*, **46**, 349–373. 10.1016/0304-3991(92)90024-e 1336234

[bb40] Leapman, R. D. & Sun, S. (1995). *Ultramicroscopy*, **59**, 71–79. 10.1016/0304-3991(95)00019-w 7571121

[bb41] Leiros, H.-K. S., McSweeney, S. M. & Smalås, A. O. (2001). *Acta Cryst.* D**57**, 488–497. 10.1107/s0907444901000646 11264577

[bb42] Leiros, H.-K. S., Timmins, J., Ravelli, R. B. G. & McSweeney, S. M. (2006). *Acta Cryst.* D**62**, 125–132. 10.1107/S0907444905033627 16421442

[bb43] Liao, H. Y. & Frank, J. (2010). *Structure*, **18**, 768–775. 10.1016/j.str.2010.05.008 PMC2923553 20637413

[bb44] Lowe, D. G. (2004). *Int. J. Comput. Vis.* **60**, 91–110.

[bb47] McEwen, B. F., Downing, K. H. & Glaeser, R. M. (1995). *Ultramicroscopy*, **60**, 357–373. 10.1016/0304-3991(95)00082-8 8525549

[bb48] McGeehan, J. E., Carpentier, P., Royant, A., Bourgeois, D. & Ravelli, R. B. G. (2007). *J. Synchrotron Rad.* **14**, 99–108. 10.1107/S0909049506043251 17211076

[bb49] McGeehan, J., Ravelli, R. B. G., Murray, J. W., Owen, R. L., Cipriani, F., McSweeney, S., Weik, M. & Garman, E. F. (2009). *J. Synchrotron Rad.* **16**, 163–172. 10.1107/S0909049509001629 PMC2651762 19240328

[bb50] McMillan, J. A. & Los, S. C. (1965). *Nature (London)*, **206**, 806–807.

[bb45] Massover, W. H. (2007). *J. Synchrotron Rad.* **14**, 116–127. 10.1107/S0909049506052307 17211078

[bb46] Massover, W. H. (2011). *Micron*, **42**, 141–151. 10.1016/j.micron.2010.05.006 20598558

[bb51] Meents, A., Gutmann, S., Wagner, A. & Schulze-Briese, C. (2010). *Proc. Natl Acad. Sci.* **107**, 1094–1099. 10.1073/pnas.0905481107 PMC2798883 20080548

[bb52] Mhaisekar, A., Kazmierczak, M. J. & Banerjee, R. (2005). *J. Synchrotron Rad.* **12**, 318–328. 10.1107/S0909049505003250 15840917

[bb53] Mikolajczyk, K., Tuytelaars, T., Schmid, C., Zisserman, A., Matas, J., Schaffalitzky, F., Kadir, T. & Gool, L. V. (2006). *Int. J. Comput. Vis.* **65**, 43–72.

[bb54] Murray, J. W., Garman, E. F. & Ravelli, R. B. G. (2004). *J. Appl. Cryst.* **37**, 513–522.

[bb55] Murray, J. W., Rudiño-Piñera, E., Owen, R. L., Grininger, M., Ravelli, R. B. G. & Garman, E. F. (2005). *J. Synchrotron Rad.* **12**, 268–275. 10.1107/S0909049505003262 15840910

[bb56] Nave, C. & Hill, M. A. (2005). *J. Synchrotron Rad.* **12**, 299–303. 10.1107/S0909049505003274 15840914

[bb57] Nowak, E., Brzuszkiewicz, A., Dauter, M., Dauter, Z. & Rosenbaum, G. (2009). *Acta Cryst.* D**65**, 1004–1006. 10.1107/S0907444909026821 PMC2733885 19690379

[bb58] O’Neill, P., Stevens, D. L. & Garman, E. (2002). *J. Synchrotron Rad.* **9**, 329–332. 10.1107/s0909049502014553 12409618

[bb59] Orlova, E. V. & Saibil, H. R. (2010). *Methods Enzymol.* **482**, 321–341. 10.1016/S0076-6879(10)82013-0 20888967

[bb60] Owen, R. L., Rudiño-Piñera, E. & Garman, E. F. (2006). *Proc. Natl Acad. Sci.* **103**, 4912–4917. 10.1073/pnas.0600973103 PMC1458769 16549763

[bb61] Paithankar, K. S. & Garman, E. F. (2010). *Acta Cryst.* D**66**, 381–388. 10.1107/S0907444910006724 PMC2852302 20382991

[bb62] Ravelli, R. B. & Garman, E. F. (2006). *Curr. Opin. Struct. Biol.* **16**, 624–629. 10.1016/j.sbi.2006.08.001 16938450

[bb63] Ravelli, R. B. & McSweeney, S. M. (2000). *Structure*, **8**, 315–328. 10.1016/s0969-2126(00)00109-x 10745008

[bb64] Ravelli, R. B. G., Nanao, M. H., Lovering, A., White, S. & McSweeney, S. (2005). *J. Synchrotron Rad.* **12**, 276–284. 10.1107/S0909049505003286 15840911

[bb65] Ravelli, R. B. G., Theveneau, P., McSweeney, S. & Caffrey, M. (2002). *J. Synchrotron Rad.* **9**, 355–360. 10.1107/s0909049502014541 12409622

[bb66] Reimer, L. & Kohl, H. (2008). *Electron Microscopy: Physics of Image Formation*, 5th ed. Berlin: Springer-Verlag.

[bb67] Rice, L. M., Earnest, T. N. & Brunger, A. T. (2000). *Acta Cryst.* D**56**, 1413–1420. 10.1107/s0907444900010039 11053839

[bb68] Royant, A., Carpentier, P., Ohana, J., McGeehan, J., Paetzold, B., Noirclerc-Savoye, M., Vernède, X., Adam, V. & Bourgeois, D. (2007). *J. Appl. Cryst.* **40**, 1105–1112.

[bb69] Royer, W. E., Sharma, H., Strand, K., Knapp, J. E. & Bhyravbhatla, B. (2006). *Structure*, **14**, 1167–1177. 10.1016/j.str.2006.05.011 16843898

[bb70] Royer, W. E., Strand, K., van Heel, M. & Hendrickson, W. A. (2000). *Proc. Natl Acad. Sci.* **97**, 7107–7111. 10.1073/pnas.97.13.7107 PMC16507 10860978

[bb71] Sage, J. T., Zhang, Y., McGeehan, J., Ravelli, R. B. G., Weik, M. & van Thor, J. J. (2011). *Biochim. Biophys. Acta.* In the press. 10.1016/j.bbapap.2011.02.012 21376143

[bb72] Saibil, H. R. (2000). *Acta Cryst.* D**56**, 1215–1222. 10.1107/s0907444900010787 10998617

[bb73] Saxton, W. O. & Frank, J. (1977). *Ultramicroscopy*, **2**, 219–227. 10.1016/s0304-3991(76)91385-1 888241

[bb74] Sindelar, C. V. & Downing, K. H. (2010). *Proc. Natl Acad. Sci.* **107**, 4111–4116. 10.1073/pnas.0911208107 PMC2840164 20160108

[bb75] Sliz, P., Harrison, S. C. & Rosenbaum, G. (2003). *Structure*, **11**, 13–19. 10.1016/s0969-2126(02)00910-3 12517336

[bb76] Snell, E. H., Bellamy, H. D., Rosenbaum, G. & van der Woerd, M. J. (2007). *J. Synchrotron Rad.* **14**, 109–115. 10.1107/S090904950604605X 17211077

[bb77] Snell, E. H., van der Woerd, M. J., Miller, M. D. & Deacon, A. M. (2005). *J. Appl. Cryst.* **38**, 69–77.

[bb78] Southworth-Davies, R. J. & Garman, E. F. (2007). *J. Synchrotron Rad.* **14**, 73–83. 10.1107/S0909049506044177 17211073

[bb79] Southworth-Davies, R. J., Medina, M. A., Carmichael, I. & Garman, E. F. (2007). *Structure*, **15**, 1531–1541. 10.1016/j.str.2007.10.013 18073104

[bb80] Stark, H. (2010). *Methods Enzymol.* **481**, 109–126. 10.1016/S0076-6879(10)81005-5 20887855

[bb81] Stenn, K. & Bahr, G. F. (1970). *J. Ultrastruct. Res.* **31**, 526–550. 10.1016/s0022-5320(70)90167-x 5425386

[bb82] Symons, M. C. R. (1999). *Prog. React. Kinet. Mech.* **24**, 139–164.

[bb83] Taylor, K. A. & Glaeser, R. M. (1976). *J. Ultrastruct. Res.* **55**, 448–456. 10.1016/s0022-5320(76)80099-8 933264

[bb85] Typke, D., Gilpin, C. J., Downing, K. H. & Glaeser, R. M. (2007). *Ultramicroscopy*, **107**, 106–115. 10.1016/j.ultramic.2006.06.005 16905258

[bb86] Utschig, L. M., Chemerisov, S. D., Tiede, D. M. & Poluektov, O. G. (2008). *Biochemistry*, **47**, 9251–9257. 10.1021/bi800574e 18690706

[bb87] Van Heel, M. (1987). *Ultramicroscopy*, **21**, 95–100. 10.1016/0304-3991(87)90078-7 12425301

[bb88] Vinogradov, S. N. & Sharma, P. K. (1994). *Methods Enzymol.* **231**, 112–124. 10.1016/0076-6879(94)31010-6 8041246

[bb89] Vulovic, M., Brandt, P., Ravelli, R. B. G., Koster, A. J., van Vliet, L. J. & Rieger, B. (2010). *International Symposium on Biomedical Imaging*, pp. 1121–1124.

[bb90] Vulovic, M., Rieger, B., van Vliet, L. J., Koster, A. J. & Ravelli, R. B. G. (2010). *Acta Cryst.* D**66**, 97–109. 10.1107/S0907444909031205 20057054

[bb92] Weik, M. & Colletier, J.-P. (2010). *Acta Cryst.* D**66**, 437–446. 10.1107/S0907444910002702 PMC2852308 20382997

[bb93] Weik, M., Ravelli, R. B., Kryger, G., McSweeney, S., Raves, M. L., Harel, M., Gros, P., Silman, I., Kroon, J. & Sussman, J. L. (2000). *Proc. Natl Acad. Sci.* **97**, 623–628. 10.1073/pnas.97.2.623 PMC15380 10639129

[bb94] Weik, M., Ravelli, R. B., Silman, I., Sussman, J. L., Gros, P. & Kroon, J. (2001). *Protein Sci.* **10**, 1953–1961. 10.1110/ps.09801 PMC2374210 11567086

[bb95] Wendler, P. & Saibil, H. R. (2010). *Biochem. Cell Biol.* **88**, 89–96. 10.1139/o09-164 20130682

[bb96] Yano, J., Kern, J., Irrgang, K. D., Latimer, M. J., Bergmann, U., Glatzel, P., Pushkar, Y., Biesiadka, J., Loll, B., Sauer, K., Messinger, J., Zouni, A. & Yachandra, V. K. (2005). *Proc. Natl Acad. Sci.* **102**, 12047–12052. 10.1073/pnas.0505207102 PMC1186027 16103362

[bb97] Zhang, X., Jin, L., Fang, Q., Hui, W. H. & Zhou, Z. H. (2010). *Cell*, **141**, 472–482. 10.1016/j.cell.2010.03.041 PMC3422562 20398923

[bb98] Zhou, Z. H. (2008). *Curr. Opin. Struct. Biol.* **18**, 218–228. 10.1016/j.sbi.2008.03.004 PMC2714865 18403197

